# Peer presence increases the prosocial behavior of adolescents by speeding the evaluation of outcomes for others

**DOI:** 10.1038/s41598-022-10115-0

**Published:** 2022-04-20

**Authors:** Nicolette J. Sullivan, Rosa Li, Scott A. Huettel

**Affiliations:** 1 Department of Management, London School of Economics and Political Science, Houghton St., London, WC2A 2AE UK; 2 Department of Psychology and Neuroscience, University of North Carolina at Chapel Hill, 332 Davie Hall, 235 E. Cameron Ave., Chapel Hill, NC 27514 USA; 3 Department of Psychology and Neuroscience, Duke University, Durham, USA

**Keywords:** Psychology, Human behaviour, Computational models

## Abstract

Peer presence can elicit maladaptive adolescent decision-making, potentially by increasing sensitivity to the rewards one receives. It remains unknown whether peer presence also increases adolescents’ sensitivity to others’ outcomes, which could have an adaptive effect in contexts allowing pro-social behaviors. Here, we combine social utility modeling and real-time decision process modeling to characterize how peer presence alters adolescents’ processing of self and other outcomes. We found that adolescents behaved selfishly when privately allocating monetary rewards for themselves and a peer in an incentive-compatible task. In peer presence, however, adolescents became more altruistic. Real-time decision process estimates collected using computer mouse tracking showed that altruistic behavior was associated with relatively earlier influence of peer-outcomes relative to self-outcomes, and that peer presence sped the influence of peer-outcomes without altering the time at which self-outcomes began to influence the decision process. Our results indicate a mechanism through which peer presence prompts greater prosocial behavior by altering how adolescents process prosocial outcomes.

## Introduction

Public health data indicate that many maladaptive behaviors peak in adolescence^1–5^, a phenomenon often attributed partially to an adolescent-specific heightened sensitivity to reward^6,7^. Peer presence has been shown to exaggerate adolescents’ reward sensitivity and, consequently, their risk-taking behaviors^8–10^. Previous studies of peer influence on adolescent decision-making have largely investigated the mechanisms underlying processing of outcomes specifically for self-rewards^8–10^ or outcomes tied to self-rewards^11,12^, yet many of adolescents’ everyday decisions affect not only themselves but also peers (see^13^ for a review). Furthermore, adolescents are motivated to earn rewards for their peers—studies have found that winning rewards for friends activate similar regions in the adolescent brain as rewards for self, just to a lesser degree^14–16^. Still, it remains unknown whether and how adolescents’ reward-processing for a peer (separately from reward-processing for self) changes while in the presence of that peer, and how this affects adolescents’ decisions when others’ outcomes are also at stake.

While early work on adolescent sensitivity to reward and peer influence tended to focus on negative consequences^1–5,8–10^, more recent work has highlighted the potential positive effects of peer sensitivity (see^17^ for review). For example, adolescents contribute more to public goods games when watched by peers^13^ and especially when those peers provided positive feedback for donating^12,13^. Furthermore, peer influence does not always prompt riskier behavior—adolescents also make more safe choices after watching a peer do so^11^—and risky behavior can also have positive outcomes, such as enrolling in a challenging class^18,19^.

Here, we consider the hypothesis that the presence of a peer increases sensitivity to others’ rewards, which in turn can facilitate prosocial behavior. We evaluate a specific mechanism for this increased sensitivity: reductions in the relative time to consider real rewards that another person will actually receive. Our study is motivated by decision process models^20–26^ that treat choices as reflecting a process of relative evidence accumulation toward one response option or another. Most such models assume that option comparison cannot begin until the qualities (i.e., values or attributes) of each option have been estimated. This onset time has been found to vary according to several characteristics of choices, including the salience of each option^27^ and attribute type^28–30^. Attributes processed earlier during option comparison receive a greater weight in choice, due to their relatively longer contribution to the decision process^28,30,31^.

Under many conditions, potential reward outcomes for the self are likely to be more concrete and relevant to the decision maker than rewards a peer may receive due to their increased psychological distance^16,32^. Because previous work has indicated that attributes at further psychological distance take longer to begin influencing the decision process^28,30,33^. If so, other-rewards would be processed relatively slower, resulting in decisions that favor advantageous inequity (i.e., peer’s outcome worse than self’s outcome) and reject disadvantageous inequity (i.e., peer’s outcome worse than self’s outcome). We propose that peer presence alters this decision process by speeding the processing of peer rewards, thus shifting people toward more altruistic choices. This may be particularly true in adolescents, who, compared to adults, are more sensitive to and spend more time with peers^8,34,35^.

**Figure 1:**
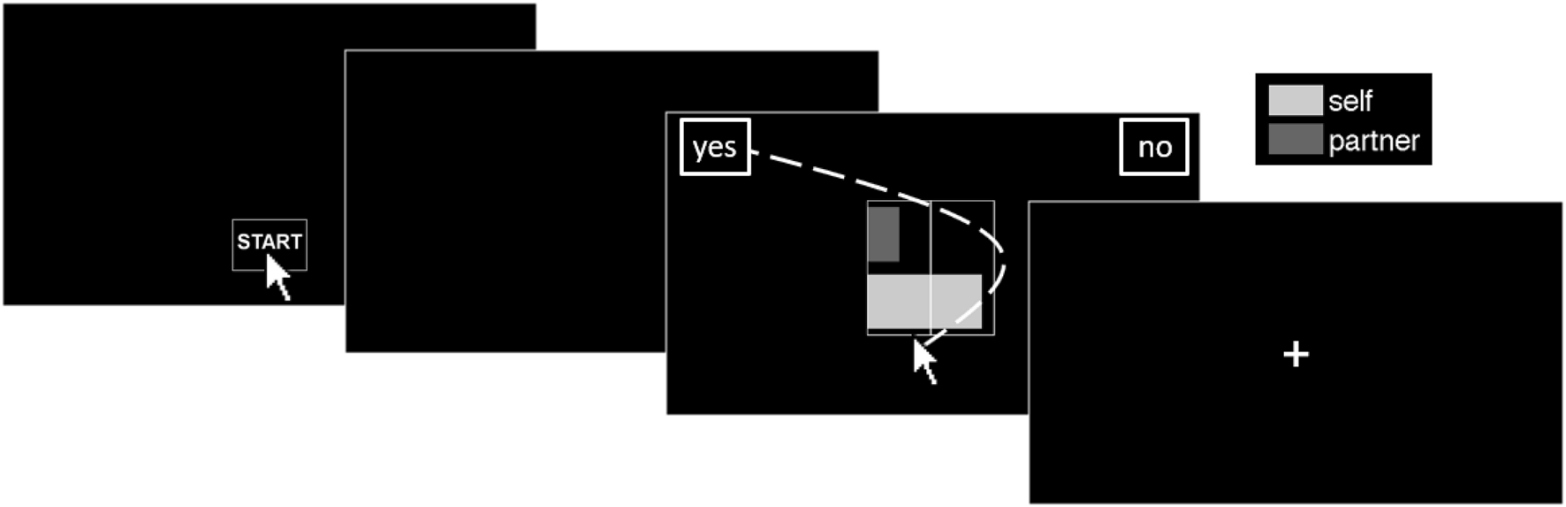
Reward allocation task. Participants used a computer mouse to accept or reject allocations of rewards for themselves (light grey bars) and their peer (dark grey bars; labeled as “partner” to the participant). Participants began each trial by clicking a START button. Once participants began moving the mouse, the current offer of between 0 and 10 points for self and 0 and 10 points for peer appeared on the center of the screen. Participants smoothly and continuously moved their mouse to the “YES” box to accept the displayed offer or the “NO” box to reject it and instead receive 5 points for self and 5 points for peer, represented by the vertical line drawn at the halfway mark of the graphical display. The trajectory of the mouse movement—and particularly, the inflection points of any change in that movement—provided information about the relative timing of when self and peer rewards influenced the decision process. Choices on randomly selected trials were converted to bonus payments for both the participant and the peer (see “Methods”).

In the current study, adolescents made incentive-compatible decisions about self-other monetary divisions within a multi-round dictator game, both alone and while watched by a peer friend who actually received the divisions allotted to the partner (Fig. 1)^36^. We quantified the influence of peer presence on social preferences using robust social utility models^37^ that capture fine-grained differences in how self- and peer-rewards affect decision-making under advantageous or disadvantageous inequity. We tracked computer mouse movements to pinpoint the times at which self and other rewards begin to contribute to decisions and how those times were altered depending on whether participants were alone or watched by a peer. The combination of social utility models and process tracing methods provides an integrated explanation for paradoxical features of adolescent decision-making: both selfish and altruistic decisions depend on the relative speed of self and other reward processing across decision contexts.

## Results

### Peer presence shifts tolerance for inequity

**Figure 2:**
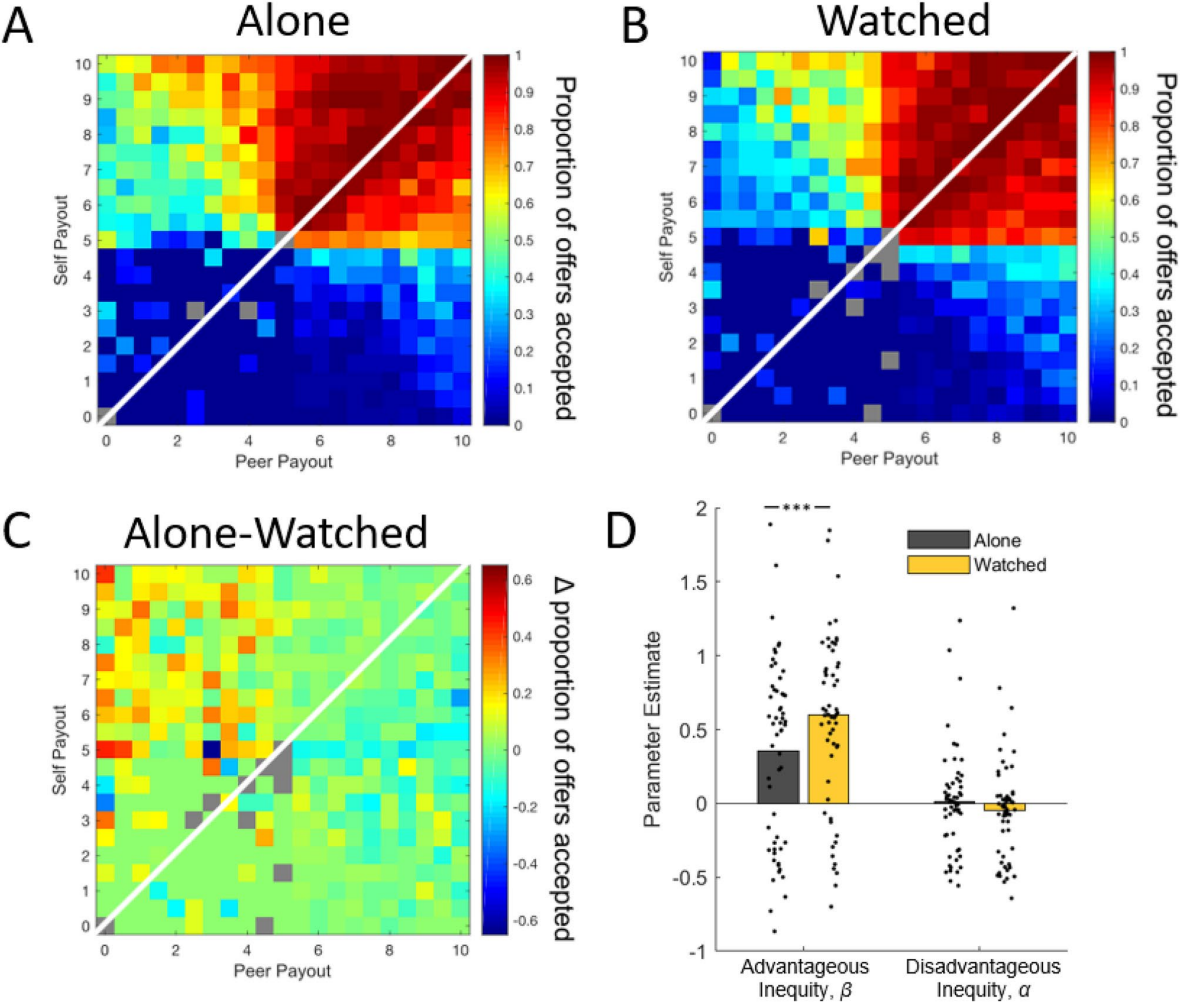
Adolescents were more averse to advantageous inequity when watched. Shown are the proportions of accepted offers for each combination of self and peer payouts when adolescents completed the task (**A**) alone and (**B**) while watched by a peer. Rejecting the offer resulted in a payout of 5-self/5-peer. Panel (**C**) depicts the change in offer acceptance when moving from completing the task Alone to being Watched by a peer. Warm colors represent more offers accepted in the Alone condition, and cooler colors represent more offers accepted in the Watched condition. For all three heatmaps, grey tiles represent offers shown fewer than three times across all participants. The white diagonal line represents a division between situations of advantageous inequity (upper left triangle) and disadvantageous inequity (lower right triangle). (**D**) Average inequity parameters when Alone and when Watched by a peer. Adolescents exhibit significantly greater βs when Watched compared to when Alone, indicating greater weight on peer outcomes (Xp) and less weight on self outcomes (Xs). Dots represent individual participants. For display purposes, the individual dots for one participant whose parameter fit outside of the [− 1, 2] range are included in the mean bars, but not displayed. *** indicates p < 0.001.

Adolescents generally accepted offers involving increased rewards to both themselves and their peer, relative to rejecting the offer in favor of 5-self/5-peer in both the Alone and Watched conditions (Fig. 2A,B; top right quadrant). They also generally rejected offers in which both self and peer would receive fewer than 5 points in both conditions (bottom left quadrant). The presence of a peer led to decreased acceptance of allocations involving *advantageous* inequity (i.e., receiving more than their peer) but increased acceptance of allocations involving *disadvantageous* inequity (i.e., receiving less than their peer; Fig. 2C). This difference by condition was particularly striking for decisions involving advantageous inequity when the participant would receive more, and their peer less, than the equitable off-screen reference (top left quadrant).

To formally characterize participants’ social preferences, we fit participant’s choices to the Fehr and Schmidt^37^ social utility model, which captures two aspects of choice behavior: a disadvantageous inequity aversion parameter (α) representing utility lost for earning less than their peer, and an advantageous inequity aversion parameter weight (β) representing utility lost for earning more than their peer. The social utility model is described below (Eq. 1), where x_s_ denotes the payout for self, x_p_ denotes the payout for peer, and U(x) denotes the participant’s utility for the trial:

1 $$U\left(x\right)= {x}_{s}- \alpha \mathrm{max}\left[{x}_{p}-{x}_{s}, 0\right]- \beta \mathrm{max}\left[{x}_{s}-{x}_{p}, 0\right]$$

We used multilevel modeling (also known as hierarchical linear modeling) to capture the random and fixed effects of α and β under Alone and Watched conditions (see “Methods”; SI).

When Alone, adolescents exhibited little disadvantageous inequity aversion (fixed effects Alone α = 0.015) but moderate advantageous inequity aversion (fixed effects Alone β = 0.34). A pairwise comparison of the estimated marginal means for Alone β versus Alone α was statistically significant (*p* = 0.0052), indicating greater advantageous inequity aversion than disadvantageous inequity aversion while Alone; Fig. 2D). One participant’s parameter fits fell outside the display range of Fig. 2D (see Fig. S2 for a figure with all participants). Bar means plotted on Fig. 2D and all statistics include this participants.

Next, we evaluated how peer presence altered inequity aversion. When Watched compared to when Alone, adolescents showed decreased aversion to disadvantageous inequity (fixed effects Watched α =  − 0.052; Alone α versus Watched α pairwise *p* = 0.041) and significantly increased aversion to advantageous inequity (fixed effects Watched β = 0.59; Alone β versus Watched β pairwise *p* = 0.0005). This indicates that the presence of a peer promoted prosociality by significantly increasing adolescent’s aversion to earning more than their peer. As in the Alone condition, the pairwise comparison of the estimated marginal means for Watched β versus Watched α was statistically significant (*p* < 0.0001), indicating greater advantageous inequity aversion than disadvantageous inequity aversion while Watched.

Participants always performed the Alone condition first to assess baseline inequity aversion without possible contaminating or priming effects of being watched by a peer, but this design does not allow us to control for task order between the Alone and Watched conditions. One concern of task order is practice effects, which we ruled out with a moving-averages analysis of behavior across each run. In the Alone condition, disadvantageous inequity aversion declines slightly over time, but advantageous inequity aversion does not (Fig. S3), and these differences were relatively small. This indicates that practice effects are unlikely to be a significant driver of the inequity aversion between the Alone and Watched conditions observed in this study. We also estimated the influence of performing the Watched condition first or last and found no statistically significant difference in either α or β (β medians first = 0.57, last = 0.61; d = − 0.06, U = 711, z = − 1.14, *p* = 0.25; α medians first = − 0.07, last = − 0.05; d = − 0.06, U = 737, z = − 0.73, *p* = 0.47).

A second concern of task order is that the adolescents completing the Watched condition last may be influenced not only by the presence of their peer but also by having just observed their peer’s choices. To rule out the effect of the latter, we reran our multilevel social utility model with just the 29 participants who performed the first Watched run immediately after the Alone condition (Watched_1_). The finding that peer presence significantly increased advantageous inequity aversion still held among the subset of participants who completed the Watched condition without knowledge of their peers’ choices (fixed effects Alone β = 0.41; fixed effects Watched_1_ β = 0.58; pairwise *p* = 0.032), while the effect of peer presence on disadvantageous inequity aversion was non-significant (fixed effects Alone α = 0.015; fixed effects Watched_1_ α = − 0.071, pairwise *p* = 0.083).

Lastly, we examined the correlations between α and β within dyads to assess whether the adolescents who performed the Watched condition last (Watched_2_) were influenced by observing their peers’ choices. Watched_2_ inequity aversion was correlated with what they had observed for advantageous, but not disadvantageous, inequity aversion (Watched_2_ r_β_, Pearson r = 0.45, *p* = 0.02; Watched_2_ r_α_, Pearson r = 0.28, *p* = 0.15). Although this could be due to friends’ shared prosocial attitudes, we note that Alone condition inequity aversion was not correlated within dyads (Alone r_β_, Pearson r = 0.16, *p* = 0.42; Alone r_α_, Pearson r = 0.06, *p* = 0.76). Due to this potential influence of watching on subsequent advantageous inequity aversion, analyses are reported with all participants as well as restricted to the Watched_1_ participants.

### Payouts to self are processed earlier than peer outcomes when alone

We next used the mouse cursor position data to estimate the time point in the decision process where self-outcomes and peer-outcomes began influencing decision processes; these time points are known as *attribute latencies* (see^28^ and SI for method). To minimize any bias in this estimation that may arise from different amplitudes of the decision weights for these monetary rewards, we normalized all time points to a proportion of each attribute’s full final amplitude (Fig. S1; see “Methods” and SI for details). We then estimated a piecewise growth model for each attribute that fits a parameter for attribute latency (i.e., the time point at which each curve in Fig. S1 diverges from zero). Non-parametric tests were used to compare latencies because self and peer latency distributions were non-normal (self, skewness = − 0.20, kurtosis = 3.96, Shapiro-Wilks W = 0.96, *p* = 0.01; peer, skewness = 1.26, kurtosis = 5.68, Shapiro-Wilks W = 0.92, *p* < 0.001).

In the Alone condition, information about self-outcomes was processed earlier than information about peer-outcomes (Fig. S4; medians, self = 668 ms, peer = 933 ms, d = − 0.82, W = 119, z = − 3.36, *p* < 0.001). This suggests that, on average, rewards to the self are processed approximately 250 ms earlier than rewards to peers when adolescents are alone (the condition that is the standard set-up for most dictator-style monetary allocation games in the lab). A control group of adult participants (see SI) was collected to assess whether this difference was a uniquely adolescent phenomenon; those results revealed that adolescents’ self-outcome latencies are statistically indistinguishable from adults’ self- and peer-outcome latencies. This indicates that adolescents’ slower processing of peer-outcomes is specific to adolescence and not an artifact of our experimental procedures.

### Peer presence increases processing speed for peers’ outcomes

**Figure 3:**
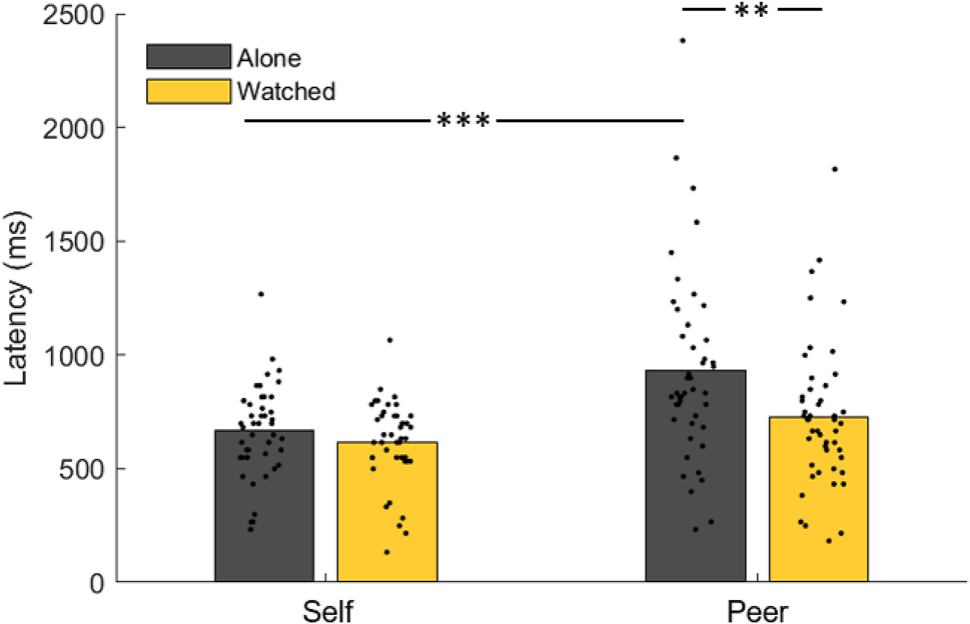
Results of reward latency estimation. Comparison of self and peer reward latencies in the alone and watched conditions. Dots represent individual participants. ** indicates p < 0.005, *** indicates p < 0.001.

Compared to the Alone condition, peer presence in the Watched condition significantly reduced the latency of processing information about rewards for peers by approximately 200 ms (Fig. 3; medians, peer alone = 933, peer watched = 726; d = 0.57, W = 618, z = 3.18, *p* = 0.001; Watched_1_ only: medians, peer alone = 922 ms, peer watched = 757 ms; d = 0.47, W = 164, z = 2.20, *p* = 0.03). Peer presence, in fact, reduced the temporal advantage held by self-outcomes in the alone condition to a non-significant difference. That is, self and peer latencies went from being statistically significantly different in the Alone condition (*p* < 0.001, see previous section) to not statistically significantly different in the Watched condition (medians, self-watched = 615 ms, peer watched = 726 ms; d = − 0.43, W = 248, z = − 1.77, *p* = 0.08; Watched_1_ participants only: medians, self = 621 ms, peer = 757 ms; d = − 0.59, W = 65.5, z = − 1.74, *p* = 0.08). Peer presence did not have a statistically significant effect upon self-reward latency (medians, alone = 668 ms, watched = 615 ms; d = 0.27, W = 328, z = 0.85, *p* = 0.40; Watched_1_ participants only: medians, alone = 674 ms, watched = 621 ms; d = 0.33, W = 79, z = 1.08, *p* = 0.28). There was not a significant attribute-by-condition interaction (ANOVA interaction F(1,177) = 3.14, *p* = 0.08; Watched_1_ only: F(1,82) = 0.02, *p* = 0.89).

### Faster relative peer outcome processing was associated with prosocial behavior

**Figure 4:**
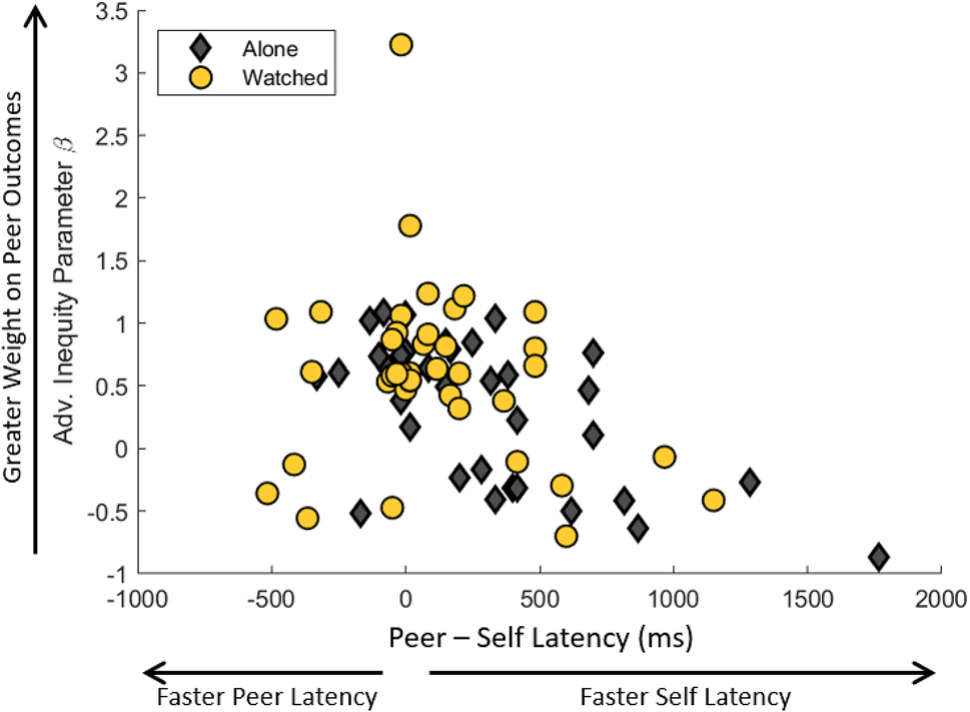
Relationship between advantageous inequity parameter β and the temporal advantage of self-reward information. The markers depict self, relative to peer, processing speeds as a function of individual best-fitting advantageous inequity parameter β, with grey diamonds representing the alone condition and yellow circles representing the watched condition. This relationship is statistically significant (p = 0.001) in a regression that also controls for condition and condition-by-latency interaction effects.

We hypothesized that differences in attribute processing speed would ramify into participants’ expressed choices, because earlier-processed attributes can influence the decision process for a longer time. We refer to this earlier processing latency as a temporal advantage^28,30^—and we examined its relationship to the model parameters^37^ for advantageous (β) and disadvantageous inequity (α). Across our adolescent participants, we observed a strong relationship between the temporal advantage of self-outcomes and prosociality. Specifically, we found that the temporal advantage for self-outcomes was correlated with prosociality under advantageous inequity (Fig. 4). To account for potential influence of condition on this relationship, our regression included condition (Watched vs. Alone) and condition-by-latency interaction terms, and an indicator for the Watched_1_ condition, none of which are statistically significant (linear regression r = 0.44, Peer—Self Latency slope = − 0.0008, 95% CI [− 0.0012 0.0003] p = 0.001; Condition slope = 0.08, 95% CI [− 0.23 0.38] p = 0.62; Interaction slope = 0.0004, 95% CI [− 0.0004 0.001] p = 0.33; Watched_1_ slope = 0.07, 95% CI [− 0.21 0.34] p = 0.64). The relationship between advantageous inequity aversion and the temporal advantage of self outcomes holds when excluding the outlier visible in Fig. 4 with a β value greater than 3 (Peer—Self Latency slope *p* = 0.0002). The temporal advantage for self-outcomes is not related to prosociality under disadvantageous inequity (Fig. S5; linear regression r = 0.13, Peer—Self Latency slope = 0.0001, 95% CI [− 1 × 10^–4^ 1 × 10^–4^], *p* = 0.41;Condition slope = − 0.02, 95% CI [− 0.20 0.16] p = 0.82; Interaction slope = − 5 × 10^–5^, 95% CI [− 0.0005 0.0004] p = 0.82; Watched_1_ slope = − 0.017, 95% CI [− 0.18 0.15] p = 0.84). Further, as this regression includes repeated measures for each participant, we performed a full mixed effects regression with condition, participant, and dyad random effects. We found that the difference in latency remained a significant predictor of prosociality under advantageous inequity (r = 0.41, Peer—Self Latency slope = 0.0007, 95% CI [0.0001 0.0003], p = 0.0002). These results provide a mechanistic explanation for prosocial allocation behavior: relatively faster processing of peer outcomes leads both to greater decision weight for peer outcomes and increased aversion to advantageous inequity.

## Discussion

This study combines incentive-compatible economic games, process tracing methods, and computational modeling to show that peer presence had selective effects on outcome processing: a greater influence on prosocial behavior under advantageous inequity than disadvantageous inequity and speeding only of outcomes for peers but not for the self. Moreover, the earlier that peers’ outcomes began to influence the decision process relative to self-outcomes, the more likely an adolescent was to consider peer payoffs in their choices. Together, these results identify a potential mechanism for adolescents’ shift in economic decisions from non-social to social contexts. Compared to aggregate choice metrics (e.g. proportion of accepted offers), the use of social utility models provided a more fine-grained measure of other-regarding preferences in adolescents^38^.

Significantly, we found that peer presence had no effect on the time at which self-outcomes entered the option comparison process—a surprising result considering that previous research on peer presence in adolescence would suggest an increased sensitivity to outcomes more globally. There are several possible explanations for this finding. It is possible that the absence of significant change between conditions reflects a “floor effect”, such that self outcomes are processed as fast as possible regardless of choice context. Alternatively, the effect of peers on adolescent reward processing could be context-specific^39^, meaning that peer presence alone was insufficient to heighten sensitivity to self-outcomes in our task, though it has been shown to increase neural response to self-outcomes in other studies^9,40^. Future studies should investigate whether relative processing speed (and subsequent choices) can also be shifted in other ways, such as by increasing the salience of self- or peer-outcomes through attentional or priming manipulations.

In this study, adolescents earned self- and peer-outcomes while being watched by the same peer who would be affected by their decisions, as is often the case in real-world peer interactions. However, this means that our results cannot differentiate between two possible alternatives: one, that peer presence increases prosociality and speeds processing of peer outcomes generally, or two, that peer presence only influences processing of outcomes for the peer that is present^41^. However, a recent study found that young adults are more generous to both strangers and close friends when being watched by non-recipient others^42^, providing evidence for the former interpretation. Our study design also cannot determine whether faster processing of peer outcomes while watched is driven by greater concern for the peer’s outcomes themselves, or by social motivations such as avoiding the social risk of seeming selfish, anticipating the benefits from appearing altruistic, or conforming to what they perceived to be more socially acceptable behavior^13,43,44^. It is possible that self-presentation concerns promote heighted attention to the rewards available to the peer, facilitating their faster processing. Future studies could disentangle these psychological motivations for giving to others by allowing participants to remain anonymous or receive credit while making observed social decisions, or varying the degree of social closeness or social status of the observing peer.

There are several limitations to our approach. First, statistically, we use a hierarchical approach to estimate inequity parameters for each condition in a single model, which allows us to account for both fixed and random effects in our parameters. Because the mouse coordinate data and choice data used for parameter fits are derived from separate inputs, our statistical comparisons of social utility parameters and processing speeds are limited because they treat those values as absolute rather than as estimates with uncertainty. Second, although previous work has drawn strong links between lab choice and behavior in more naturalized contexts^45^, we acknowledge that decision processes observed in the laboratory do not always align with those observed in the field^46^, so we are unable to conclude how well this relatively abstract task reflects real-world social interaction. Third, we are limited in that our study primarily focuses on adolescent behavior. Future work should fully replicate this study in populations of younger children and adults in order to understand the full developmental trajectory of prosocial behavior under various contexts, and to determine whether adolescents are uniquely susceptible to peer presence^47^.

Early studies of adolescent susceptibility to peer influence generally focused on how peer presence prompted maladaptive risk-taking behaviors^8–10,48^. Here, we find that peer presence instead can have a salutary effect on adolescents’ behavior. This happens specifically in the context of altruistic choice, in which peers become more averse to inequity when in the presence of an affected peer. With access to opportunities to engage in prosocial behavior, the presence of peers can be leveraged as a force for societal good, such as participating in group volunteering projects or engaging in collective political advocacy^49^.

## Methods

### Participants

Fifty-eight adolescents (U.S. high school juniors and seniors) were enrolled in the study (see SI for target sample size calculations); one participant who missed too many catch trials was excluded prior to data analysis. Thus, our final sample comprised 57 adolescents (mean age = 17.2 years; range 15.7–18.8 years; SD = 0.56 years; 38 F) who completed the experimental conditions in a within-participant design. We used a cultural definition of adolescence such that all participants were still enrolled in high school and had not yet reached full independence from their parents/guardians^43^. Participants were drawn from a community participant pool: 4 Hispanic or Latino, 53 non-Hispanic or Latino; 15 Asian, 7 Black or African American, 30 White or Caucasian, 3 reporting more than one race, 2 declined to respond. Data from a complementary adult sample of 29 participants are reported in the SI.

All participants and parents/legal guardians of adolescent participants gave written informed consent in a protocol approved by Duke University’s Institutional Review Board. Participants were informed at the time of recruitment and consent that their peer would potentially observe them at some point in the study, but the instructions provided no advance information about repeating the task in Watched and Alone conditions. Thus, participants were blinded to the experimental conditions until the moment of participation.

Adolescent participants enrolled in the study with a same-gendered peer who also completed the study at the same time, and their decisions influenced each other’s payments. Dyads were self-reported to be close friends on the Friendship Qualities Scale^50^ with 74% of participants rating closeness of at least 4 out of 5 (closeness response mean = 3.76, SD = 0.95). Participants first completed 300 trials of the task in separate rooms (*Alone* condition); this allowed assessment of baseline inequity aversion without the potential contaminating influence of the Watched condition (i.e., eliminating the potential confounding factor that adolescents watching their peer donate to them would influence their behavior). Next, both adolescents of the dyad were moved into the same testing room (*Watched* condition), and one adolescent completed another 300 trials while the peer watched from a nearby chair. Finally, adolescents in each dyad switched the watching/task roles for up to 300 more trials of the task [47 completed all 300 trials in the Watched condition, 8 completed as many trials as time allowed (range 131–262 trials) and 2 did not have time to complete the Watched condition]. During the Watched condition, participants were instructed not to communicate with each other, but the task-watcher was asked to record on a private worksheet how much he or she agreed with their peer’s choices. This procedure was implemented not only to ensure that the task-watcher remained engaged, but also to emphasize that the peer was watching (and evaluating) their choices.

### Task

Participants used a computer mouse to accept or reject point allocations for themselves and their peer. Each trial began with “START” at the bottom center of the screen. When the participant clicked “START”, the screen went black. Once the participant began moving the mouse vertically, the current offer appeared in the center and boxes containing “YES” and “NO” appeared in the top corners of the screen (randomly left/right counterbalanced across trials). Participants were instructed to smoothly and continuously move their mouse to “YES” to accept the displayed offer or to “NO” to reject it; if the offer was rejected, then they and their peer would each receive 5 points. Offers were depicted using bars, with full bars representing 10 points (see Fig. 1). A vertical line at the halfway mark represented the reference 5/5 allocation. The top/bottom position of the self and peer bars was randomly counterbalanced across trials. Stimuli were presented using the Psychophysics Toolbox^51^ for MATLAB. All procedures and methods were implemented in accordance with guidelines and regulations provided by Duke University’s Institutional Review Board.

Offers ranged from 0 to 10 points for self or peer in half-point increments, with 5/5 and 0/0 offers omitted. For each participant, we drew a unique set of offers from this range such that a person of average preferences (determined by pilot testing) would be close to indifferent on 1/3 of trials, accept 1/3 of trials, and reject 1/3 of trials. Catch trials of equal self/peer payouts that should be readily accepted (e.g. 8/8) or rejected (e.g. 4/4) were included to ensure that participants were attending to and understood the task. One adolescent was excluded from analyses for missing more than 1 catch trial per run and failing a binomial test of greater than chance performance on catch trials.

For incentive compatibility, at the end of the study, one randomly selected trial from each run was converted into dollars using a predetermined exchange rate revealed to the participant after the task (38 cents/point if a participant completed two runs; 75 cents/point if a participant only completed one run). Thus, participants did not know the exact monetary value of the offers, which controlled for potential individual differences in money valuation.

### Choice models

We fit participants’ choices to the Fehr–Schmidt social utility model^37^ using a multilevel approach (see SI) that allowed us to examine the effect of Alone and Watched condition while accounting for our repeated measures design^52–54^. The social utility model is described below (Eq. 1), where x_s_ denotes the payout for self, x_p_ denotes the payout for peer, and U(x) denotes the participant’s utility for the trial):

1 $$U\left(x\right)= {x}_{s}- \alpha \mathrm{max}\left[{x}_{p}-{x}_{s}, 0\right]- \beta \mathrm{max}\left[{x}_{s}-{x}_{p}, 0\right]$$

The parameter α represents the amount of utility that is lost under disadvantageous inequity (peer > self), while the parameter β represents the amount of utility that is lost under advantageous inequity (self > peer). The original Fehr–Schmidt model restricted alpha > beta > 0, under the assumption that people were averse to inequity and that they were more averse to disadvantageous inequity than to advantageous inequity. In our multilevel approach, we did not make this assumption and thus imposed no restrictions upon the parameter estimates. Our resulting parameter estimates were highly accurate at correctly predicting participants’ choices: an average of 90.5% correct for the Alone condition (range 76.6–98.2%) and 91.8% correct for the Watched condition (range 75.6–99.0%).

### Mouse tracking analysis

The cursor’s position was tracked with a temporal resolution equal to the screen refresh rate (60 Hz). At each time point, the cursor angle was normalized such that 0° represented direct upward movements, −45° represented movements directly toward the left option, and 45° represented movements directly toward the right option. Trials in which participants did not make continuous, goal-directed mouse movements, were excluded (see SI).

We used multiple linear regression to estimate (for each participant and condition independently) how self (*S*) and peer (*P*) outcomes (in terms of points) influenced the cursor angle at each time point. For timepoint t, this regression (Eq. 2) used the differences in rewards associated with the left and right options to predict the mouse cursor’s angle of movement between time t and t + 1, *Θ*_*t*_.

2 $${\theta }_{t}= {\beta }_{0,t}+ {\beta }_{1,t }\left({S}_{right-left}\right)+ {\beta }_{2,t}\left({P}_{right-left}\right)+{\epsilon }_{t}$$

Next, we normalized each timepoint’s self and peer coefficients from Eq. (3) to be proportional to the coefficient’s full final weight, β_N_ (Eq. 3)

3 $${\beta }_{t}^{*}=\frac{{\beta }_{t}}{{\beta }_{N}}$$

Lastly, we fit a logistic growth model (Eq. S6) to the coefficient curves (plotted in Fig. S1) to identify the first time point at which self and peer coefficients became significantly greater than zero (that is, began to drive the mouse cursor; see SI).

## Supplementary Information

Supplementary Information.

## Data Availability

All data that support the findings of this study, and the stimuli used to collect the data, as well as complete analysis scripts, are available here: https://osf.io/9nxsw/.
