# Author Correction: Peer presence increases the prosocial behavior of adolescents by speeding the evaluation of outcomes for others

**DOI:** 10.1038/s41598-022-17425-3

**Published:** 2022-08-01

**Authors:** Nicolette J. Sullivan, Rosa Li, Scott A. Huettel

**Affiliations:** 1 Department of Management, London School of Economics and Political Science, Houghton St., London, WC2A 2AE UK; 2 Department of Psychology and Neuroscience, University of North Carolina at Chapel Hill, 332 Davie Hall, 235 E. Cameron Ave., Chapel Hill, NC 27514 USA; 3 Department of Psychology and Neuroscience, Duke University, Durham, USA

Correction to: *Scientific Reports* 10.1038/s41598-022-10115-0, published online 20 April 2022

The original version of this Article contained errors, where parameters for every participant varied by on average 1 × 10^–6^. Consequently, numeric results in the original Article were incorrect.

As a result, in the Results under the subheading, ‘Peer presence shifts tolerance for inequity.’,

“When Alone, adolescents exhibited little disadvantageous inequity aversion (fixed effects Alone α = 0.018) but moderate advantageous inequity aversion (fixed effects Alone β = 0.35). A pairwise comparison of the estimated marginal means for Alone β versus Alone α was statistically significant (*p* = 0.0033), indicating greater advantageous inequity aversion than disadvantageous inequity aversion while Alone; Fig. 2D).”

now reads:

“When Alone, adolescents exhibited little disadvantageous inequity aversion (fixed effects Alone α = 0.015) but moderate advantageous inequity aversion (fixed effects Alone β = 0.34). A pairwise comparison of the estimated marginal means for Alone β versus Alone α was statistically significant (*p* = 0.0052), indicating greater advantageous inequity aversion than disadvantageous inequity aversion while Alone; Fig. 2D).”

And,

“Next, we evaluated how peer presence altered inequity aversion. When Watched compared to when Alone, adolescents showed marginally decreased aversion to disadvantageous inequity (fixed effects Watched α = − 0.046; Alone α versus Watched α pairwise *p* = 0.05) and significantly increased aversion to advantageous inequity (fixed effects Watched β = 0.60; Alone β versus Watched β pairwise *p* = 0.0004).”

now reads:

“Next, we evaluated how peer presence altered inequity aversion. When Watched compared to when Alone, adolescents showed decreased aversion to disadvantageous inequity (fixed effects Watched α = − 0.052; Alone α versus Watched α pairwise *p* = 0.041) and significantly increased aversion to advantageous inequity (fixed effects Watched β = 0.59; Alone β versus Watched β pairwise *p* = 0.0005).”

Additionally,

“We also estimated the influence of performing the Watched condition first or last and found no statistically significant difference in either α or β (β medians first = 0.57, last = 0.63; d = − 0.08, U = 714, z = − 1.28, *p* = 0.20; α medians first = − 0.07, last = − 0.04; d = −0.08, U = 741, z = − 0.86, *p* = 0.39).”

now reads:

“We also estimated the influence of performing the Watched condition first or last and found no statistically significant difference in either α or β (β medians first = 0.57, last = 0.61; d = − 0.06, U = 711, z = − 1.14, *p* = 0.25; α medians first = − 0.07, last = − 0.05; d = −0.06, U = 737, z = − 0.73, *p* = 0.47).”

Furthermore,

“The finding that peer presence significantly increased advantageous inequity aversion still held among the subset of participants who completed the Watched condition without knowledge of their peers’ choices (fixed effects Alone β = 0.41; fixed effects Watched_1_ β = 0.58; pairwise *p* = 0.032), while the effect of peer presence on disadvantageous inequity aversion was non-significant (fixed effects Alone α = 0.015; fixed effects Watched α =  − 0.071, pairwise *p* = 0.083).”

now reads:

“The finding that peer presence significantly increased advantageous inequity aversion still held among the subset of participants who completed the Watched condition without knowledge of their peers’ choices (fixed effects Alone β = 0.41; fixed effects Watched_1_ β = 0.58; pairwise *p* = 0.032), while the effect of peer presence on disadvantageous inequity aversion was non-significant (fixed effects Alone α = 0.015; fixed effects Watched_1_ α = − 0.071, pairwise *p* = 0.083).”

And,

“Watched_2_ inequity aversion was correlated with what they had observed for advantageous, but not disadvantageous, inequity aversion (Watched_2_ rβ = 0.44, *p* = 0.02; Watched_2_ rα = 0.23, *p* = 0.23). Although this could be due to friends’ shared prosocial attitudes, we note that Alone condition inequity aversion was not correlated within dyads (Alone rβ = 0.16, *p* = 0.40; Alone rα = 0.03, *p* = 0.86).”

now reads:

“Watched_2_ inequity aversion was correlated with what they had observed for advantageous, but not disadvantageous, inequity aversion (Watched_2_ rβ, Pearson r = 0.45, *p* = 0.02; Watched_2_ rα, Pearson r = 0.28, *p* = 0.15). Although this could be due to friends’ shared prosocial attitudes, we note that Alone condition inequity aversion was not correlated within dyads (Alone rβ, Pearson r = 0.16, *p* = 0.42; Alone rα, Pearson r = 0.06, *p* = 0.76).”

Under the subheading, ‘Payouts to self are processed earlier than peer outcomes when alone.’,

“To minimize any bias in this estimation that may arise from different amplitudes of the decision weights for these monetary rewards, we normalized all time points to a proportion of each attribute’s full final amplitude (Fig. S2; see “Methods” and SI for details). We then estimated a piecewise growth model for each attribute that fits a parameter for attribute latency (i.e., the time point at which each curve in Fig. S2 diverges from zero).”

now reads:

“To minimize any bias in this estimation that may arise from different amplitudes of the decision weights for these monetary rewards, we normalized all time points to a proportion of each attribute’s full final amplitude (Fig. S1; see “Methods” and SI for details). We then estimated a piecewise growth model for each attribute that fits a parameter for attribute latency (i.e., the time point at which each curve in Fig. S1 diverges from zero).”

And,

“In the Alone condition, information about self-outcomes was processed earlier than information about peer outcomes (Fig. S4; medians, self = 672 ms, peer = 933 ms, d = − 0.82, W = 119, z = − 3.36, *p* < 0.001).”

now reads:

“In the Alone condition, information about self-outcomes was processed earlier than information about peer outcomes (Fig. S4; medians, self = 668 ms, peer = 933 ms, d = − 0.82, W = 119, z = − 3.36, *p* < 0.001).”

Under the subheading ‘Peer presence increases processing speed for peers’ outcomes.’,

“Compared to the Alone condition, peer presence in the Watched condition significantly reduced the latency of processing information about rewards for peers by approximately 200 ms (Fig. 3; medians, peer alone = 933, peer watched = 726; d = 0.57, W = 618, z = 3.18, *p* = 0.001; Watched_1_ only: medians, peer alone = 904 ms, peer watched = 731 ms; d = 0.50, W = 204, z = 2.52, *p* = 0.01). Peer presence, in fact, reduced the temporal advantage held by self-outcomes in the alone condition to a non-significant difference. That is, self and peer latencies went from being statistically significantly different in the Alone condition (*p* < 0.001, see previous section) to not statistically significantly different in the Watched condition (medians, self-watched = 615 ms, peer watched = 726 ms; d = −0.43, W = 248, z = − 1.77, *p* = 0.08; Watched_1_ participants only: medians, self = 628 ms, peer = 731 ms; d = − 0.44, W = 80.5, z = − 1.49, *p* = 0.14). Peer presence did not have a statistically significant effect upon self-reward latency (medians, alone = 668 ms, watched = 615 ms; d = 0.27, W = 328, z = 0.85, *p* = 0.40; Watched_1_ participants only: medians, alone = 682 ms, watched = 628 ms; d = 0.33, W = 86, z = 0.93, *p* = 0.35). There was not a significant attribute-by condition interaction (ANOVA interaction F(1,177) = 3.14, *p* = 0.08; Watched_1_ only: F(1,94) = 0.35, *p* = 0.56).”

now reads:

“Compared to the Alone condition, peer presence in the Watched condition significantly reduced the latency of processing information about rewards for peers by approximately 200 ms (Fig. 3; medians, peer alone = 933, peer watched = 726; d = 0.57, W = 618, z = 3.18, *p* = 0.001; Watched_1_ only: medians, peer alone = 922 ms, peer watched = 757 ms; d = 0.47, W = 164, z = 2.20, *p* = 0.03). Peer presence, in fact, reduced the temporal advantage held by self-outcomes in the alone condition to a non-significant difference. That is, self and peer latencies went from being statistically significantly different in the Alone condition (*p* < 0.001, see previous section) to not statistically significantly different in the Watched condition (medians, self-watched = 615 ms, peer watched = 726 ms; d = −0.43, W = 248, z = − 1.77, *p* = 0.08; Watched_1_ participants only: medians, self = 621 ms, peer = 757 ms; d = − 0.59, W = 65.5, z = − 1.74, *p* = 0.08). Peer presence did not have a statistically significant effect upon self-reward latency (medians, alone = 668 ms, watched = 615 ms; d = 0.27, W = 328, z = 0.85, *p* = 0.40; Watched_1_ participants only: medians, alone = 674 ms, watched = 621 ms; d = 0.33, W = 79, z = 1.08, *p* = 0.28). There was not a significant attribute-by condition interaction (ANOVA interaction F(1,177) = 3.14, *p* = 0.08; Watched_1_ only: F(1,82) = 0.02, *p* = 0.89).”

In the Methods under the subheading ‘Choice models.’,

“Our resulting parameter estimates were highly accurate at correctly predicting participants’ choices: an average of 90.3% correct for the Alone condition (range 76.6–98.2%) and 91.8% correct for the Watched condition (range 75.6–99.0%).”

now reads:

“Our resulting parameter estimates were highly accurate at correctly predicting participants’ choices: an average of 90.5% correct for the Alone condition (range 76.6–98.2%) and 91.8% correct for the Watched condition (range 75.6–99.0%).”

Lastly, the Data availability section contained an error, where

“All data that support the findings of this study, and the stimuli used to collect the data, as well as complete analysis scripts, will be deposited in an OSF repository upon publication.”

now reads:

“All data that support the findings of this study, and the stimuli used to collect the data, as well as complete analysis scripts, are available here: https://osf.io/9nxsw/.”

The original Article has been corrected.

