# Supplementary material for: Peer presence increases the prosocial behavior of adolescents by speeding the evaluation of outcomes for others
Source: Sci Rep. 2022 Apr 20;12:6477. doi: 10.1038/s41598-022-10115-0 (PMC9021292; doi:10.1038/s41598-022-10115-0)
Supplement: Supplementary file 1 — Supplementary Information. [file 41598_2022_10115_MOESM1_ESM.docx]

**Supplementary Methods**

**Participants**

***Adolescent sample***

The targeted sample size (60) was determined based on a pilot dataset that included a similar binary choice task. First, we calculated the difference in self and other latencies (the times at which self and other outcomes are processed; see “Mouse tracking: Data Analysis” below) in a pilot dataset using the same task (N=32) which generated an approximate sample size of 50 (one-sample t-test of self vs. other latencies vs. 0, sampsizepwr function in MATLAB and a p < 0.05 threshold and power = 0.99). Because latencies are often non-normally distributed, we added 10 additional participants to this size estimate ^1^. Our final recruited sample was 58 adolescents.

***Adult Alone comparison sample***

Twenty-nine adult participants drawn from the Duke University community enrolled in the study with a same-gendered peer who completed a separate study. One adult was excluded from the study for missed catch trials, resulting in a final sample of 28 adults (mean age 22.7 years; range = 18.3 to 28.3; SD = 0.56; 12F; 1 Hispanic or Latino, 27 non-Hispanic or Latino; 12 Asian, 2 Black or African American, 13 White or Caucasian, 1 more than one race) who each completed a single 300-trial run Alone, as well as a demographics questionnaire. We note that though there was some age overlap in our two participant groups, we chose to use a cultural definition of adolescence by labeling 18-year-old high-school students as adolescents and those 18-years-old and older who live independently from their parents as adults ^2^.

***Model Selection***

We fit choice data to the Fehr-Schmidt (1999) social utility model using multilevel models that fit fixed and random effects for our α and β parameters of interest (AIC = 20418; BIC = 20443). We then tested whether the model was significantly improved by including interactions between the parameters of interest and Alone or Watched condition (AIC = 19096; BIC = 19213). Because the latter model yielded the lowest AIC and BIC, we proceeded with that model for further analyses.

***Mouse tracking supplemental methods: exclusion criteria***

*A priori* chosen exclusion criteria were that trials with reaction times (RTs) greater than two standard deviations above a subject’s mean, and trials in which the mouse trajectory crossed the midline of the x-axis (i.e. an invisible vertical line bisecting the screen) more than three times (a mean of 9.2% excluded trials per subject).

***Mouse tracking supplemental methods: regression correction***

We identified the timepoint at which this β*_t_ for each attribute became greater than zero; that is, when that attribute began to gain some portion of its final attribute weighting. To do this, we modeled the increase in proportion of final attribute weight on cursor using a generalized logistic function, using a population growth equation e.g., ^3,4,5^. Unlike a traditional growth model, our function begins at zero, so the model was modified to a piecewise function (Eq. 5). This function includes a parameter (t*) that specifies the time begins (i.e., the time at which the function changes from β*_t_ = 0 to β*_t_ > 0). We use this as our estimate of the time at which an attribute begins to influence the mouse cursor, and therefore the time at least by which the brain must have processed this value information, which we label as *attribute latency*. This extensive procedure was implemented to minimize the possibility that the final timepoint’s outcome coefficient – that is, the weight participants placed on self- or peer-outcomes – did not artificially bias latency estimation see reference ^6 for further discussion of this concern^. However, we should also note that all results hold using the uncorrected estimation time used in ^6^. Processing speeds for self and peer outcomes were unable to be estimated for 11 and 12 participants respectively, so those participants were excluded from analyses of processing speeds.

| $t<t^{*}, \beta_{t}^{*}=0$  $t\geq t^{*}, \beta_{t}^{*}=\frac{1}{1+be^{-kt}}$ | [S6] |
| --- | --- |

**Supplementary Results**

***Adolescent Self- and Peer-Outcome Latency Compared to Young Adult Control Group***

To assess whether earlier self, relative to peer, payout latencies are a uniquely adolescent phenomenon or if it carried into adulthood, we collected an additional dataset of young adults to perform the Alone condition of this task. For the young adult control group alone, self- and peer-outcome latencies did not differ (median self-outcome 741 ms, peer-outcome, 687 ms; d = 0. 32, U = 702.50, z = 0.45, p = 0.66). Adolescents and the control group of young adults exhibited statistically indistinguishable processing speed for outcomes for self (adolescent median = 668 ms, young adult median = 670 ms; d = -0.32, z = -0.48, p = 0.63). However, adolescents were markedly slower than adults at processing peers’ outcomes (adolescent median = 933 ms, young adult median = 687 ms; d = 0. 61, U = 478.50, z = 3.09, p = 0.002). To statistically test this age-by-payout-type interaction, we performed an ANOVA predicting latency using latency type (self or peer) as a within-subjects variable and age group as a between-subjects variable using fitrm and ranova in MATLAB. This interaction term was significant (F(1,54) = 9.17, p = .004).

***Faster relative processing of peer- outcomes associated with larger decision weights for peer outcomes***

For each adolescent participant, we estimated the weight participants placed on self and peer outcomes, without imposing any additional model assumptions. To do so, we estimated decision weights for the influence of self and peer outcomes (right–left) on choice (1= right, 0 = left), with one logistic regression per participant. Next, we estimated the relationship between the relative weight of self and peer outcomes (self–peer) on relative self and peer outcome latencies (self–peer). We found that earlier processing of peer, relative to self, latencies were associated with a larger weight placed on peer, relative to self, outcomes in choice (Fig. S6. linear regression r = 0.44, slope = -0.0009, 95% CI = [-2x10^-3^ -3x10^-4^] *p* = 0.005).

**Supplementary Figures**

**
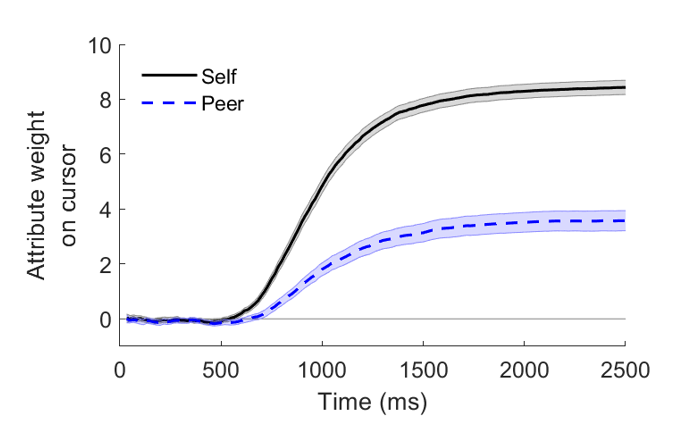
**

**Fig. S1**. Influence of self and peer outcomes on mouse trajectories. Results from the analyses exploring the mean effect of the relative self-outcome of the right item over the left item (self_right_ – self_left_) and the relative peer outcomes of the right item over the left item (peer_right_ – peer_left_), collapsed across all adolescent alone and watched conditions. Error bands denote standard errors.


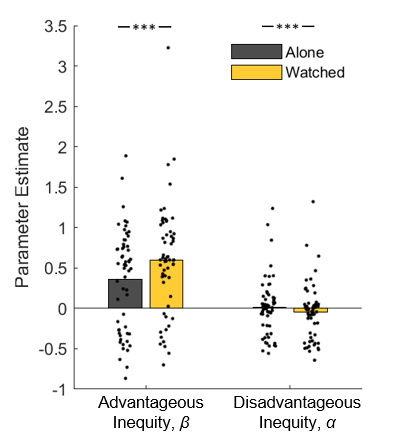


**Fig. S2.** Average inequity parameters when alone and when watched by a peer. Adolescents exhibit significantly greater βs and αs when watched compared to when alone, indicating greater weight on peer outcomes and less weight on self outcomes. Dots represent individual participants.

**
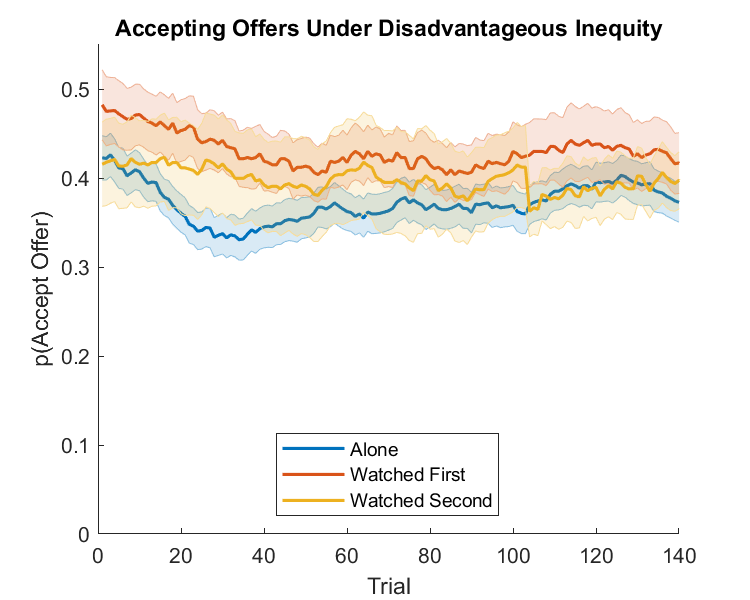

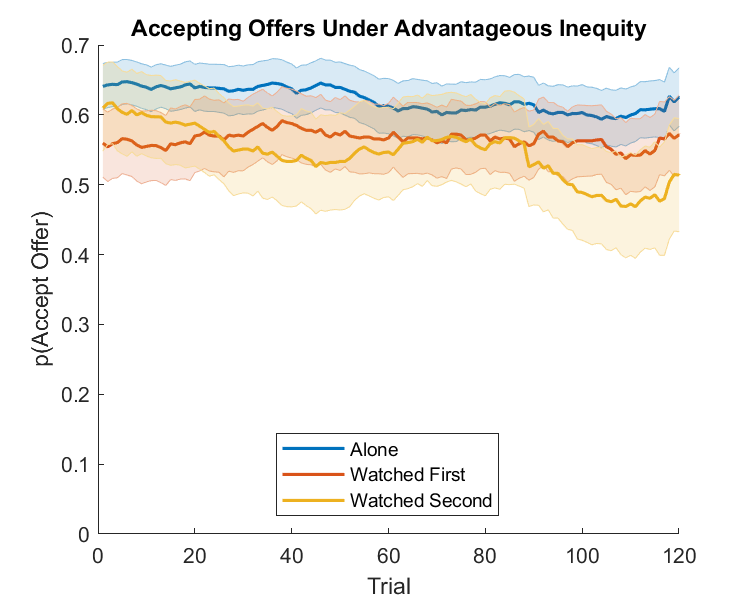
**

**Fig. S3.** Twenty-five trial moving average of proportion offers accepted under conditions of disadvantageous (left) and advantageous (right) inequity.

**
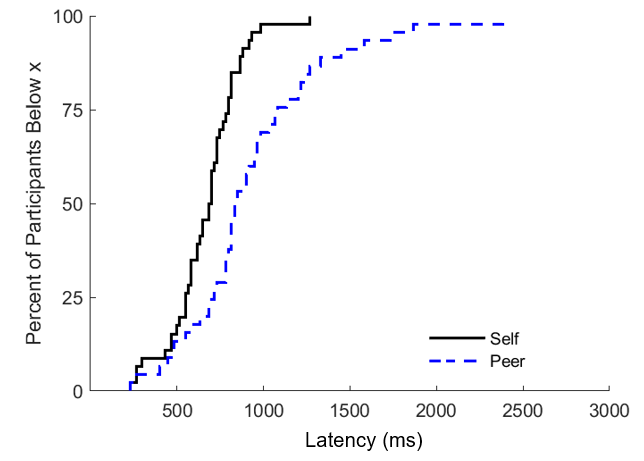
**

**Fig. S4.** Results of outcome latency estimation in the alone condition. The distribution of self and peer outcome latencies are shown in this cumulative distribution graph for the watched condition.


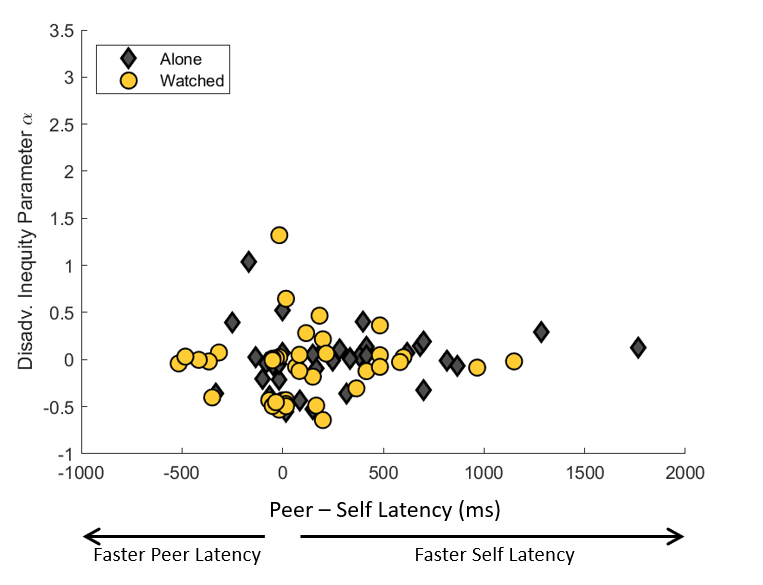


**Fig. S5**. The relationship between disadvantageous inequity parameter α and the computational advantage of self-outcome information. The markers depict self, relative to peer, processing speeds as a function of individual best-fitting advantageous inequity parameter α, with grey diamonds representing the alone condition and yellow circles representing the watched condition This relationship is not statistically significant (*p* = 0.41) in a regression that also controls for condition and condition-by-latency interaction effects.

**Supplement References**

1 Lehmann, E. L. *Nonparametrics : Statistical Methods Based on Ranks, Revised*. 76-81 (Pearson Education, 1998).

2 Crone, E. A. & Dahl, R. E. Understanding adolescence as a period of social-affective engagement and goal flexibility. *Nat Rev Neurosci* **13**, 636-650, doi:10.1038/nrn3313 (2012).

3 Richards, F. J. *A Flexible Growth Model for Empirical Use*. Vol. 10 (1959).

4 Tsoularis, A. & Wallace, J. Analysis of logistic growth models. *Mathematical biosciences* **179**, 21-55 (2002).

5 Zwietering, M. H., Jongenburger, I., Rombouts, F. M. & van 't Riet, K. Modeling of the bacterial growth curve. *Applied and environmental microbiology* **56**, 1875-1881 (1990).

6 Sullivan, N., Hutcherson, C., Harris, A. & Rangel, A. Dietary self-control is related to the speed with which attributes of healthfulness and tastiness are processed. *Psychological science* **26**, 122-134, doi:10.1177/0956797614559543 (2015).
